# Decline of instrumental activities of daily living is a risk factor for nutritional deterioration in older adults: a prospective cohort study

**DOI:** 10.1186/s12877-023-04185-6

**Published:** 2023-08-09

**Authors:** Koutatsu Nagai, Takuya Komine, Miho Ikuta, Mako Gansa, Ryota Matsuzawa, Kayoko Tamaki, Hiroshi Kusunoki, Yosuke Wada, Shotaro Tsuji, Kyoko Sano, Ken Shinmura

**Affiliations:** 1 Department of Physical Therapy, School of Rehabilitation, Hyogo Medical University, 1-3-6 Minatojima, Chuo-ku, Kobe, Hyogo 650-8530 Japan; 2 Department of Physical Therapy, Hyogo Rehabilitation Center, 1080,Akebono-cho,Nishi-ku, Kobe, Hyogo, 651-2181 Japan; 3 Department of Therapy, Hakuhokai Central Hospital, 4-23-1 Higashisonodacho, Amagasaki, Hyogo, 661-0953 Japan; 4 Department of Therapy, Amagasaki Central Hospital, 1-12-1 Sioe, Amagasaki, Hyogo, 661-0976 Japan; 5 Department of General Medicine, Hyogo Medical University, 1-1 Mukogawa, Nishinomiya, Hyogo 663-8501 Japan; 6 Department of Internal Medicine, Osaka Dental University, 1-8 Kuzuha-hanazono, Hirakata, Osaka 573-1121 Japan; 7 Roppou Clinic, 1-465 Imamori, Toyooka, Hyogo 668-0851 Japan; 8 Department of Orthopaedic Surgery, Hyogo Medical University, 1-1 Mukogawa, Nishinomiya, Hyogo 663-8501 Japan; 9 Department of Therapy, Takarazuka Rehabilitation Hospital, 2-22 Tsuruno-so, Takarazuka, Hyogo 665-0833 Japan

**Keywords:** Functional capacity, IADL, Nutrition, MNA, Older adults

## Abstract

**Background:**

The association between functional capacity and the subsequent risk of nutritional deterioration is yet to be understood. The purpose of this study was to elucidate the relationship between functional capacity, comprising instrumental activities of daily living (IADL), intellectual activity, and social function, and future decline in nutritional status.

**Methods:**

The current study is a two-year prospective cohort study. A total of 468 community-dwelling older adults without nutritional risks were enrolled. We used the Mini Nutritional Assessment Screening Form. Functional capacity, including IADL, intellectual activity, and social function, was assessed using the Tokyo Metropolitan Institute of Gerontology Index of Competence at baseline. The nutritional status was reassessed at a 2-year follow-up. Risk ratios (RR) of functional capacity for the incidence of nutritional decline were estimated.

**Results:**

Low functional capacity was significantly associated with future deterioration of nutritional status (RR 1.12, 95% confidence interval [CI] 1.02–1.25). Of the subdomains of functional capacity, IADL decline (adjusted RR 2.21, 95% CI 1.18–4.13) was an independent risk factor for the incidence of nutritional risk. Intellectual and social activities were not significant.

**Conclusion:**

Decline in functional capacity, especially IADL, is a risk factor for future deterioration in nutritional status. Further studies are required to elucidate the effect of interventions for IADL decline on maintaining nutritional status in older adults.

**Supplementary Information:**

The online version contains supplementary material available at 10.1186/s12877-023-04185-6.

## Background

Good nutrition is a key factor in the aging process and a considerable contributor to healthy aging, helping to maintain favorable health and reducing the risk of chronic disease[1, 2]. Malnutrition is associated with significantly increased morbidity and impaired functional ability[3]. Consequently, individuals who are malnourished or at risk of malnutrition exhibit higher mortality rates compared to those who are well-nourished [4, 5]. Earlier studies conducted in older adults demonstrated that 5–10% of them were malnourished and 30–50% were categorized as at risk of malnutrition, even in community settings[6–8]. In addition, its prevalence in nursing homes or hospital settings is higher than that in community[8]. Malnutrition is a serious problem that threatens the health of many older adults.

Nutritional decline has been linked to several chronic diseases, depressive symptoms, loneliness, and functional dependency among community-dwelling older adults[9–12]. Systematic reviews examining risk factors for malnutrition in older adults have indicated that polypharmacy, cognitive decline, oral dysphagia, poor appetite, and edentulousness were also related to the deterioration of nutritional status[13, 14]. Identifying risk factors for worsening nutritional status enables healthcare professionals to provide preventive approaches to malnutrition in older populations.

Functional capacity is a crucial aspect of maintaining independent living among older adults. Lawton’s model conceptualizes functional capacity as comprising three subdomains: instrumental activities of daily living (IADL), intellectual activity, and social function[15]. Decreased IADLs, including shopping, meal preparation, and public transportation, may negatively contribute to the nutritional status of older people. Intellectual activities, including reading newspapers or books, and interest in health may also associated with maintaining the nutritional status in older adults.

However, it remains unclear whether functional capacity is associated with future nutritional risk. Furthermore, it is unclear which subdomains of functional capacity are closely associated with nutritional status. A previous review examining the association between IADL and malnutrition concluded that sufficient evidence was unavailable due to the lack of studies[14]. This understanding would facilitate the formulation of a preventive strategy aimed at reducing the nutritional risk or prevalence of malnutrition among older adults. The purpose of this study was to elucidate the association between functional capacity and a future decline in nutritional status and to determine which domains of functional capacity predict it.

## Methods

### Study design and participants

This study was a prospective cohort study. Baseline data were collected from 2015 to 2017. The population’s average age in the area was higher than the national average, with 31.4% of individuals being aged 65 or older. Participants were recruited for the study through community advertising, placement of posters at hospital, and verbal announcements made by medical personnel. The 2-year follow-up assessment was performed to determine the frequency of decline in the nutritional status.

The targeted population were individuals who were not at nutritional risk or malnourished according to the Mini Nutritional Assessment Screening Form (MNA-SF). The inclusion criteria consisted of: (1) age ≥ 65 years and (2) normal nutritional status. The exclusion criteria were as follows: (1) Moderate to severe cognitive impairment assessed by a Mini-Mental State Examination (MMSE) score of < 21 [16] and (2) incomplete data.

During the initial stage, a total of 844 individuals participated in the baseline assessment, out of which 724 met the inclusion criteria (120 out of 844 were at nutritional risk or were malnourished). Twelve people were excluded due to cognitive decline or missing data. Of 712 people, 244 were excluded due to the missing information regarding nutritional status at the follow-up assessment. The 468 participants were included in the analysis.

#### Nutritional assessment

A clinical dietitian assessed the nutritional status using the MNA-SF tool, which comprises six inquiries covering appetite, weight loss, mobility, psychological stress, neuropsychological issues, and body mass index (BMI). The scoring of MNA-SF ranges from 0 to 14, with patients falling into three categories: “normal nutritional status” (scores 12–14), “at nutritional risk” (scores 8–11), or “malnourishment” (scores 0–7)[2, 17].

#### Functional capacity

At the baseline assessment, the participants’ functional capacity was assessed using the Tokyo Metropolitan Institute of Gerontology Index of Competence (TMIG-IC) questionnaire[18]. This questionnaire includes three competence subdomains, namely IADL, intellectual activity, and social function. Each subdomain is scored on a scale ranging from 0 to 5 for IADL, 0 to 4 for intellectual activity, and 0 to 4 for social function. The IADL subdomain assesses the participant’s ability to utilize public transport independently, shop for essential items, prepare meals independently, pay bills, and manage banking without assistance. The intellectual activity subdomain evaluates the participant’s capability to complete pension forms, read newspapers, read books or magazines, and exhibit an interest in news stories or television programs that pertain to health-related subjects. The social function subdomain assesses the participant’s ability to visit friends at their residence, advise family or friends, ability to visit ill friends, and communicate with younger individuals. The total maximum score for the TMIG-IC questionnaire was 13 points, with higher scores indicating better functioning. The definition of impairments in each functional capacity was based on scores that were lower than the maximum possible score[19].

### Other variables

The age, sex, comorbidities, and education of the participants were self-reported. The Barthel index was used to evaluate the function of basic ADLs[20]. Additionally, the Geriatric Depression Scale (GDS) [21] was employed to assess depressive symptoms. Venous blood was collected during the interviews, and serum was prepared from the blood.

### Outcome measure

The main outcome was the nutritional status assessed using the MNA-SF score. Participants were re-assessed for nutritional status during the 2-year follow-up, and were classified as either maintaining a normal nutritional status (scores 12–14) or declined nutritional status, which indicates nutritional risk or malnourishment (scores < 12), to determine the temporal association between functional capacity and nutritional status. As an additional analysis, we focused on the subdomains of functional capacity, consisting of IADL, intellectual activity, and social activity. Then, we conducted an exploratory analysis to determine which sub-items in the MNA mainly deteriorated.

### Statistical analysis

We assessed the differences in baseline characteristics between the maintained nutritional status group and the declined nutritional status group (MNA-SF scores < 12) during the 2-year follow-up using appropriate statistical tests based on the nature of the variables. Selection bias was assessed by comparing baseline characteristics in participants who were successfully followed up and non-followed after 2 years.

We used a modified Poisson regression model[22] to calculate the risk ratio (RR) and 95% confidence interval (CI) of functional capacity for the decline in nutritional status. First, functional capacity assessed by TMIG-IC, as a continuous variable, was included as an independent variable. The TMIG-IC score was then transformed so that higher scores indicated lower function. Second, three subdomains of functional capacity, IADL, intellectual activity, and social activity, were entered as independent variables as binomial data[19] in the different models. As adjusted variables, age and sex were selected as basic demographics (Model 2). For the adjusted variables, age and sex were selected as basic demographics (Model 2). Additionally, the MMSE score, GDS score, number of comorbidities, and medications were selected as potential confounders that could influence the association between functional capacity and nutritional status [23–28] (Model 3). Then, we assessed these associations in subitems of the MNA-SF to determine the detailed association between functional capacity and MNA-SF. The change in the MNA score during the 2-year follow-up was then entered as the dependent variable. IBM SPSS ver. 24 (IBM Japan Ltd., Tokyo, Japan) was utilized to analyze the data. The level of statistical significance was set at P < 0.05.

## Results

Participants’ characteristics at baseline are shown according to the maintained nutritional status group and declined nutritional status group during the 2-year follow-up (Table 1). At the 2-year follow-up, 49 (10%) of 468 declined nutritional status and 419 (90%) were maintained (Table 1). Of the participants with nutritional decline, 45 were categorized as at risk and 4 as malnutrition. The frequencies of participants with decreased scores on each question in the MNA-SF at the 2-year follow-up were as follows: Appetite: 26 (5.6%), Weight loss: 42 (9.0%), Mobility: 1 (0.2%), Psychological stress: 26 (5.6%), Neuropsychological problems or acute disease: 3 (0.6%), and BMI: 75 (16%). According to the results of the assessment of selection bias (see Appendix Table), non-follow-up participants had low cognitive function, intellectual activity, and physical functioning.

**Table 1:** Baseline differences between normal nutrition and nutritional decline groups during the 2-year follow-up

	Overall (n = 468)	Normal nutrition (n = 419)	Nutritional decline (n = 49)	P-value
Age, y, mean (SD)	72.8 (5.7)	72.8 (5.8)	72.7 (5.3)	0.899
Woman, n (%)	304 (65.0)	269 (64.2)	35 (71.4)	0.316
Height, cm, mean (SD)	155.9 (8.3)	155.9 (8.2)	155.5 (8.8)	0.755
Body weight, kg, mean (SD)	56.4 (9.4)	56.9 (9.1)	52.2 (10.7)	0.001
BMI, kg/m2, mean (SD)	23.1 (2.7)	23.3 (2.6)	21.5 (3.1)	< 0.001
Medication, n, median (IQR)	1.0 (0–3)	1.0 (0–3)	1.0 (0–3)	0.409
Comorbidities				
Hypertension, n (%)	217 (46.4)	197 (47.0)	20 (40.8)	0.410
Diabetes, n (%)	56 (12.0)	50 (11.9)	6 (12.2)	0.949
Kidney disease, n (%)	20 (4.3)	18 (4.3)	2 (4.1)	0.650
Cardiovascular disease, n (%)	34 (7.3)	31 (7.4)	3 (6.1)	0.513
Osteoporosis, n (%)	53 (11.3)	42 (10.0)	11 (22.4)	0.009
Stroke, n (%)	6 (1.3)	6 (1.4)	0 (0)	0.513
Cancer, n (%)	38 (8.1)	34 (8.1)	4 (8.2)	0.581
Number of comorbidities	1 (0–1)	1 (0–1)	1 (0-1)	0.498
GDS, score, median (IQR)	0.0 (0–2)	0.0 (0–2)	1.0 (0–3)	0.043
Serum albumin, g/dl, mean (SD)	4.3 (0.3)	4.3 (0.3)	4.3 (0.2)	0.449
MMSE, median (IQR)	29 (27–30)	29 (27–30)	29 (28–30)	0.898
Education, y, Median (IQR)	12 (12–14)	12 (12–14)	12 (12–14)	0.365
Barthel index, score, median (IQR)	100 (100–100)	100 (100–100)	100 (100–100)	0.142
TMIG-IC, score, median (IQR)	13.0 (13–13)	13 (13–13)	13 (12–13)	0.098
IADL, n (%)				0.011
5	414 (88.5)	376 (89.7)	38 (77.6)	
3–4	53 (11.3)	42 (10.0)	11 (22.5)	
0–2	1 (0.2)	1 (0.2)	0 (0)	
Intellectual activity, n (%)				0.048
4	404 (86.3)	366 (87.4)	38 (77.6)	
2–3	28 (6.0)	25 (5.9)	3 (6.1)	
0–1	36 (7.7)	28 (6.7)	8 (16.3)	
Social function, n (%)				0.213
4	397 (84.8)	358 (85.4)	39 (79.6)	
2–3	69 (14.8)	59 (14.0)	10 (20.4)	
0–1	2 (0.4)	2 (0.5)	0 (0)	

SD: standard deviation, IQR: interquartile range, BMI: body mass index, GDS: Geriatric Depression Scale, TMIG-IC: Tokyo Metropolitan Institute of Gerontology Index of Competence, MMSE: Mini-Mental State Examination, IADL: instrumental activities of daily living, BI: Barthel index

χ2 test or Fisher exact test for proportions, Student t-test for parametric variables, and Mann– Whitney U test for non-parametric variables

By comparing the baseline characteristics between each nutritional group during the 2-year follow-up, there were significant differences in body weight, BMI, osteoporosis, GDS, IADL, and intellectual activity (Table 1).

Modified Poisson regression showed that low functional capacity was a significant risk factor for deterioration of nutritional status during the 2-year follow-up (crude RR 1.12, 95% CI 1.02–1.25, fully adjusted RR 1.12, 95% CI 1.01-1.23) (Table 2). A secondary analysis using the subdomains of functional capacity showed that reduced IADL (crude RR 2.22, 95% CI 1.21–4.08, fully adjusted RR 2.21, 95% CI 1.18–4.13) was an independent risk factor for the development of nutritional risk. The decreased intellectual activity was not significant slightly for the future decline in nutritional status (crude RR 1.83, 95% CI 0.97–3.39, fully adjusted RR 1.79, 95% CI 0.97–3.32), and declined social activity was also not associated with it (crude RR, 1.43; 95% CI 0.75–2.74, fully adjusted RR 1.28, 95% CI, 0.638–2.55). In our Poisson regression model with binomial independent variables, the calculated statistical power for examining the association of IADL decline, intellectual activity decline, and social role decline with nutritional risk was 0.607, 0.411, and 0.147, respectively.

**Table 2:** Risk ratios for the decline of nutritional status after 2 years according to baseline functional capacity

	Model 1			Model 2			Model 3		
	RR	95% CI	P-value	RR	95% CI	P-value	RR	95% CI	P-value
TMIG-IC score	1.12	1.02–1.25	0.020	1.12	1.02–1.23	0.022	1.12	1.01–1.23	0.034
IADL decline	2.22	1.21–4.08	0.010	2.25	1.22–4.14	0.009	2.21	1.18–4.13	0.013
Intellectual activity decline	1.83	1.00–3.39	0.056	1.83	0.99–3.40	0.054	1.79	0.97–3.32	0.065
Social function decline	1.43	0.75–2.74	0.275	1.43	0.74–2.76	0.292	1.28	0.64–2.55	0.490

Model 1: Crude model

Model 2: Adjusted for age and gender

Model 3: In addition to covariates in Model 2, comorbidities, medication, GDS, and MMSE were added

The subdomains of TMIG-IC were analyzed with different statistical models

TMIG-IC score was transformed before the entry so that higher scores indicated lower function. Thus, the risk ratio represents the risk of worsening nutritional status for a one-point worsening of the TMIG score

IADL decline, intellectual activity decline, and social function decline were incorporated into the model as binomial variables

RR: Risk ratio, CI: confidence interval, TMIG-IC: Tokyo Metropolitan Institute of Gerontology Index of Competence, GDS: Geriatric depression scale, MMSE: Mini-Mental State Examination, IADL: instrumental activities of daily living

Of the sub-items in the MNA-SF, weight loss was the most sensitive outcome related to functional capacity (Table 3).

**Table 3:** Risk ratios for the decline of each question in MNA–SF after 2 years according to baseline functional capacity

	Model 1			Model 2			Model 3		
	RR	95% CI	P-value	RR	95% CI	P-value	RR	95% CI	P-value
**A. Appetite**									
TMIG-IC score	1.00	1.00–1.00	0.427	1.00	1.00–1.01	0.465	1.00	0.99–1.01	0.589
IADL decline	1.02	0.97–1.07	0.409	1.02	0.97–1.07	0.389	1.02	0.97–1.06	0.472
Intellectual activity decline	1.01	0.97–1.05	0.517	1.01	0.97–1.05	0.576	1.00	0.96–1.04	0.896
Social function decline	1.01	0.97–1.05	0.603	1.01	0.97–1.04	0.752	1.00	0.97–1.04	0.892
**B. Weight loss**									
TMIG-IC score	1.01	1.00–1.03	0.043	1.01	1.00–1.03	0.048	1.01	1.00–1.03	0.064
IADL decline	1.07	1.00–1.16	0.065	1.07	1.00–1.16	0.063	1.07	0.99–1.16	0.078
Intellectual activity decline	1.08	1.01–1.16	0.034	1.08	1.01–1.16	0.041	1.07	1.00–1.15	0.058
Social function decline	1.06	1.00–1.13	0.058	1.06	0.99–1.12	0.075	1.05	0.99–1.12	0.103
** C. Mobility**									
TMIG-IC score	1.00	1.00–1.00	0.323	1.00	1.00–1.00	0.323	1.00	1.00–1.00	0.326
IADL decline	1.01	0.99–1.03	0.315	1.01	0.99–1.03	0.313	1.01	0.99–1.02	0.292
Intellectual activity decline	1.00	1.00–1.00	0.317	1.00	1.00–1.00	0.320	1.00	0.99–1.00	0.303
Social function decline	1.01	0.99–1.02	0.316	1.01	0.99–1.02	0.315	1.01	1.00–1.01	0.300
**D. Psychological stress**									
TMIG-IC score	1.01	0.99–1.02	0.446	1.01	0.99–1.02	0.503	1.00	0.99–1.02	0.593
IADL decline	1.04	0.95–1.13	0.407	1.04	0.95–1.14	0.376	1.04	0.95–1.14	0.417
Intellectual activity decline	1.02	0.99–1.00	0.601	1.02	0.99–1.00	0.648	1.02	0.94–1.10	0.692
Social function decline	1.01	0.99–1.00	0.761	1.01	0.99–1.00	0.893	1.00	0.93–1.07	0.887
**E. Neuropsychological problems or acute disease**									
TMIG-IC score	1.00	1.00–1.00	0.102	1.00	1.00–1.00	0.109	1.00	1.00–1.00	0.135
IADL decline	1.00	0.99–1.00	0.102	1.00	0.99–1.00	0.103	0.99	0.99–1.00	0.120
Intellectual activity decline	1.00	0.99–1.00	0.102	1.00	0.99–1.00	0.107	0.99	0.98–1.00	0.134
Social function decline	1.00	0.99–1.00	0.102	1.00	0.99–1.00	0.110	1.00	0.99–1.00	0.136
** F. BMI**									
TMIG-IC score	1.01	0.99–1.03	0.207	1.01	0.99–1.03	0.227	1.01	0.99–1.03	0.216
IADL decline	1.03	0.93–1.13	0.582	1.03	0.94–1.13	0.545	1.03	0.94–1.14	0.517
Intellectual activity decline	1.07	0.97–1.18	0.177	1.07	0.97–1.18	0.188	1.08	0.98–1.19	0.122
Social function decline	1.07	0.98–1.16	0.142	1.06	0.97–1.16	0.171	1.06	1.00–1.15	0.209

Model 1: Crude model

Model 2: Adjusted for age and gender

Model 3: In addition to covariates in Model 2, comorbidities, medication, GDS, and MMSE were added

The risk ratio represents the risk of worsening nutritional status for a one-point worsening of the TMIG score

The change in MNA score during the 2-year follow-up was entered as the dependent variable

IADL decline, intellectual activity decline, and social function decline were incorporated into the model as binomial variables

The subdomains of TMIG-IC were analyzed with different statistical models

MNA-SF: Mini Nutritional Assessment Screening Form, RR: risk ratio, CI: confidence interval, TMIG-IC: Tokyo Metropolitan Institute of Gerontology Index of Competence, IADL: instrumental activities of daily living, BMI: body mass index, GDS: Geriatric Depression Scale, MMSE: Mini-Mental State Examination

## Discussion

This prospective cohort study investigated the temporal relationship between functional capacity and future deterioration of nutritional status. A decline in functional capacity was related to the development of nutritional risks. Among the subdomains of functional capacity, the decline in IADL was a significant risk factor for the deterioration of nutritional status. Of the sub-items in the MNA-SF, weight loss appeared to be the most sensitive factor.

Functional capacity, especially IADL, was associated with future deterioration in nutritional status. Although cross-sectional relationships have been reported, it was unclear whether the decline in IADL preceded the deterioration in nutritional status. IADL includes shopping for daily necessities and meal preparation, paying bills, and using public transport. Some earlier studies have cross-sectionally shown the relationship between limited IADL and nutritional risk[11, 29–31], and contributions of IADL to good nutritional status have been mentioned. The ability to shop is an important aid in maintaining good nutrition, and difficulty with transportation is a significant barrier [29, 30, 32]. Difficulty in meal preparation can be a key factor in malnutrition, especially in healthy older adults[31, 32]. In the present study, weight loss seemed to be the sensitive subitem of the MNA-SF. Considering these results, one could hypothesize that a decrease in functional capacity, including IADL, contributes to weight loss, thereby leading to a decline in nutritional status. However, the significant association we observed between functional capacity and weight loss became somewhat less significant in the fully adjusted model. As such, we were unable to reach a definitive conclusion.

The temporal association between intellectual activity and nutritional status was not statistically confirmed, although consistent trends were observed. Cognitive decline affects daily functional status, which can lead to dependence and decreased oral intake[33] and is also associated with nutritional status[11]. The present study focused on the independent risk of intellectual activity for nutritional status by adjusting for cognitive function, and it was not significant. Intellectual activities include the capacity to gather information related to health topics, which represents health literacy[34]. A higher health literacy contributes to better nutritional quality[35]. Hence, we hypothesized that intellectual activity could be related to a reduction in the risk of nutritional decline. It seems difficult to conclude the direct association of that activity with future nutritional status based on the current results.

Social functioning did not contribute to preventing the decline in nutritional status. When the social aspects of eating are considered, food consumption may increase, thereby improving nutritional status[36]. Loss of family or friends is negatively associated with intake[37]. Fewer friends, loneliness, and the death of a spouse increase the risk of protein-energy malnutrition[14]. Despite the relationship between social aspects and nutritional factors, no such association was found in this study. The questionnaire used in the current study included only limited aspects of social factors, such as visiting friends and advice to someone, which might bring different results to those of earlier studies.

Malnutrition is associated with the incidence of IADL disability, including basic ADL[38]. Our results, coupled with the association, implicate a hypothetical model wherein nutritional status and IADL could be interinfluenced. The current investigation, being an observational study, might have bias and unknown confounding variables; as a result, it is important to interpret the causal connections between IADL and malnutrition with caution. Nevertheless, the possibility that maintaining IADL or assistance as functional capacity can contribute to the prevention of nutritional deterioration seems logical and should be considered.

The current study has some limitations. First, a few participants dropped out of the 2-year follow-up assessments. Indeed, non-followed participants presented lower cognitive function and intellectual activity than the follow-up participants, which may have involved a selection bias. However, this difference was marginal. Therefore, we considered that the risk of serious selection bias was not high. Second, we treated nutritional risk (MNA-SF scores from 8 to 11) or malnutrition (scores from 0 to 7) as indicators of nutritional deterioration, without using a full version of MNA. Therefore, the current results cannot definitively conclude that decreased functional capacity is associated with the onset of malnutrition. Third, the statistical power by the post-hoc analysis was not high, which suggesting that larger sample size could have potentially yielded more significant results. Hence, future studies with larger sample sizes may help to strengthen these findings. Fourth, since this study is observational, not intervention, we can’t r assert any causal associations.

## Conclusions

Our study underscores that a decline in functional capacity, especially IADL, is a risk factor for future deterioration of nutritional status. Among the sub-items of the MNA-SF, weight loss appears to be a sensitive domain. Further studies are required to elucidate the effects of interventions for IADL decline on maintaining nutritional status in older adults.

### Electronic supplementary material

Below is the link to the electronic supplementary material.

Supplementary Material 1

## Data Availability

The datasets analyzed during the current study are available from the corresponding author on reasonable request.

## Abbreviations

IADL: instrumental activities of daily living
RR: Risk ratio
CI: confidence interval
MNA-SF: Mini Nutritional Assessment Screening Form
MMSE: Mini-Mental State Examination
BMI: body mass index
TMIG-IC: Tokyo Metropolitan Institute of Gerontology Index of Competence
GDS: Geriatric Depression Scale
