# Supplementary material for: Decline of instrumental activities of daily living is a risk factor for nutritional deterioration in older adults: a prospective cohort study
Source: BMC Geriatr. 2023 Aug 9;23:480. doi: 10.1186/s12877-023-04185-6 (PMC10413727; doi:10.1186/s12877-023-04185-6)
Supplement: Supplementary file 1 — Supplementary Material 1 [file 12877_2023_4185_MOESM1_ESM.docx]

Supplementary Material

| Appendix Table. Baseline differences between followed and non-followed participants during the 2-year follow-up | | | |
| --- | --- | --- | --- |
|  | Followed participants (n = 468) | Non-followed participants  (n = 244) | p-value |
| Age, y, mean (SD) | 72.8 (5.7) | 73.0 (6.3) | 0.649 |
| Woman, n (%) | 304 (65.0) | 171 (70.1) | 0.168 |
| Height, cm, mean (SD) | 155.9 (8.3) | 154.9 (8.1) | 0.113 |
| Body weight, mean (SD) | 56.4 (9.4) | 55.9 (8.7) | 0.456 |
| BMI, kg/m2, mean (SD) | 23.1 (2.7) | 23.2 (2.6) | 0.620 |
| Medication, n, median (IQR) | 1.0 (0–3) | 1.0 (0-3) | 0.664 |
| Comorbidities |  |  |  |
| Hypertension, n (%) | 217 (46.4) | 110 (45.1) | 0.744 |
| Diabetes, n (%) | 56 (12.0) | 33 (13.5) | 0.551 |
| Kidney disease, n (%) | 20 (4.3) | 4 (1.6) | 0.065 |
| Cardiovascular disease, n (%) | 34 (7.3) | 17 (7.0) | 0.884 |
| Osteoporosis, n (%) | 53 (11.3) | 26 (10.7) | 0.787 |
| Stroke, n (%) | 6 (1.3) | 7 (2.9) | 0.116 |
| Cancer, n (%) | 38 (8.1) | 15 (6.1) | 0.341 |
| MNA-SF, score, median (IQR) | 14 (13–14) | 14 (13–14) | 0.705 |
| GDS, score, median (IQR) | 0.0 (0–2) | 0 (0-3) | 0.128 |
| Serum albumin, g/dl, mean (SD) | 4.3 (0.3) | 4.4 (0.3) | 0.066 |
| MMSE, median (IQR) | 29.0 (27–30) | 29.0 (26–30) | 0.028 |
| Education, y, Median (IQR) | 12 (12–14) | 12 (12–12) | 0.255 |
| Barthel index, score, median (IQR) | 100 (100–100) | 100 (100–100) | 0.557 |
| TMIG-IC, score, median (IQR) | 13.0 (13–13) | 13.0 (12–13) | 0.075 |
| IADL, n (%) |  |  | 0.270 |
| 5 | 414 (88.5) | 209 (85.7) |  |
| 3–4 | 53 (11.3) | 34 (14.0) |  |
| 0–2 | 1 (0.2) | 1 (0.4) |  |
| Intellectual activity, n (%) |  |  | 0.033 |
| 4 | 404 (86.3) | 195 (79.9) |  |
| 2–3 | 28 (6.0) | 26 (10.7) |  |
| 0–1 | 36 (7.7) | 23 (9.4) |  |
| Social function, n (%) |  |  | 0.080 |
| 4 | 397 (84.8) | 195 (79.9) |  |
| 2–3 | 69 (14.8) | 44 (18.1) |  |
| 0–1 | 2 (0.4) | 5 (2.0) |  |
| SD: standard deviation, IQR: interquartile range, BMI: body mass index, MNA-SF: Mini Nutritional Assessment Screening Form, GDS: Geriatric Depression Scale, TMIG-IC: Tokyo Metropolitan Institute of Gerontology Index of Competence, MMSE: Mini-Mental State Examination, IADL: instrumental activities of daily living, BI: Barthel index. | | | |
| χ2 test or Fisher exact test for proportions, Student t-test for parametric variables, and Mann–Whitney U test for non-parametric variables. | | | |
